# Online health information behaviour and its association with statin adherence in patients with high cardiovascular risk: A prospective cohort study

**DOI:** 10.1177/20552076241241250

**Published:** 2024-03-21

**Authors:** Hooi Min Lim, Chirk Jenn Ng, Adina Abdullah, Mahmoud Danee, Jacques Raubenheimer, Adam G. Dunn

**Affiliations:** 1 Department of Primary Care Medicine, Faculty of Medicine, 37447 Universiti Malaya, Kuala Lumpur, Malaysia; 2 Department of Research, 68752 SingHealth Polyclinics, Singapore, Singapore; 3 121579 Duke-NUS Medical School, Singapore, Singapore; 4 Department of Social and Preventive Medicine, Faculty of Medicine, 37447 Universiti Malaya, Kuala Lumpur, Malaysia; 5 Biomedical Informatics and Digital Health, Faculty of Medicine and Health, School of Medical Sciences, 4334 The University of Sydney, Sydney, Australia

**Keywords:** consumer health informatics, information-seeking behaviour, health literacy, medication adherence, cardiovascular risk

## Abstract

**Objective:**

Statins are effective for preventing cardiovascular disease. However, many patients decide not to take statins because of negative influences, such as online misinformation. Online health information may affect decisions on medication adherence, but measuring it is challenging. This study aimed to examine the associations between online health information behaviour and statin adherence in patients with high cardiovascular risk.

**Methods:**

A prospective cohort study involving 233 patients with high cardiovascular risk was conducted at a primary care clinic in Malaysia. Participants used a digital information diary tool to record online health information they encountered for 2 months and completed a questionnaire about statin necessity, concerns and adherence at the end of the observation period. Data were analysed using structural equation modelling.

**Results:**

The results showed that 55.8% (130 of 233 patients) encountered online health information. Patients who actively sought online health information (91 of 233 patients) had higher concerns about statin use (*β* = 0.323, *p* = 0.023). Participants with higher concern about statin use were also more likely to be non-adherent (*β* = -0.337, *p* < 0.001). Patients who actively sought online health information were more likely to have lower statin adherence, mediated by higher concerns about statin use (indirect effect, *β* = -0.109, *p* = 0.048).

**Conclusions:**

Our results suggest that patients with higher levels of concern about statins may be actively seeking online information about statins, and their concerns might influence how they search, what they find, and the potential to encounter misinformation. Our study highlights the importance of addressing patients’ concerns about medications to improve adherence.

## Introduction

Statins are effective in preventing cardiovascular disease (CVD), and long-term use of statins is recommended for patients with high cardiovascular risk.^1,2^ A critical challenge with their use in the community is non-adherence. Adherence to statins ranges from 30% to 60% globally,^3^ and less than half of people prescribed statins achieve their low-density lipoprotein cholesterol level targets.^4^ Statin non-adherence is a complex process influenced by patients, healthcare providers, and system-level factors. Common factors related to non-adherence include negative perceptions of statin use such as fear of side effects, doubts about the necessity of statins, and lack of perceived effectiveness.^5,6^

The health information that patients encounter may influence the decisions they make about medications.^7^ People encounter health information from a variety of sources including consultations with healthcare providers, people they trust, and both online and offline sources.^8^ Online health information behaviour can be categorised into active information-seeking and passive receipt of health information.^9^ People actively search for online health information for a specific purpose or may be exposed to health information unintentionally during their daily activities. Online health information may influence how people understand diseases and decide on treatments.^10,11^ Current systematic review suggests that online health information-seeking is one of the factors influencing medication adherence.^12^ However, the association is mixed, as some studies indicate that online health information-seeking is linked to improved medication adherence, while others report lower adherence levels. This variability is dependent on factors such as specific health conditions and types of medication. In the context of statin adherence, studies examining the association between online health information behaviour and medication adherence have been scarce. A survey in Denmark reported that patients who search for health information from the media before their visits to physicians are more likely to discontinue statins.^13^ An experimental study in undergraduate students showed that participants are more likely to recommend statins to an older relative if they are exposed to information related to the benefits of statins.^14^ Further research needs to be conducted in patients with high cardiovascular risk.

Relevant studies in the area measure direct associations of online health information with medication adherence,^12^ but do not examine how other factors that might mediate the relationship between online health information behaviour and adherence. Several health behaviour models including the Health Belief Model^15^ and the Theory of Planned Behaviour^16^ have been used to explain why patients decide to engage in health behaviour. Specifically for medication adherence, the Necessity-Concern Framework (NCF)^17^ was developed to assess the beliefs of patients and their medication adherence. According to the NCF, patients are more likely to adhere to a medication when their perceived need for treatment (necessity) is higher than their worries about the treatment side effects (concern).^18^ The NCF has been applied in several medication adherence studies related to cardiovascular diseases including adherence to lipid-lowering medications,^19^ antihypertensive medications,^20^ and medications for ischaemic heart diseases.^21^ Our previous qualitative study suggested that patients who encounter poor-quality online health information are concerned about statin side effects.^22^ We know of no studies that examine whether concern and necessity of statin use mediate the relationship between online health information and statin adherence.

Another major challenge with research in the area is the bluntness and heterogeneity in how exposures to online health information are measured.^12^ Most studies are cross-sectional and use a questionnaire asking participants to recall frequency and sources of online health information.^23,24^ In what follows, we use an information diary to avoid common issues such as recall bias, and capture more detailed information about the health information that study participants search for or are exposed to, as well as capturing online and offline sources.^25^

The aim of this study was to examine the associations between online health information behaviour and statin adherence among patients with high cardiovascular risk. We examined whether concern and necessity of statin use mediate the relationship between online health information behaviour and statin adherence, and whether trust in doctors and eHealth literacy moderate the relationship between online health information behaviour and statin adherence.

## Methods

### Research model and hypotheses

**Figure 1.**
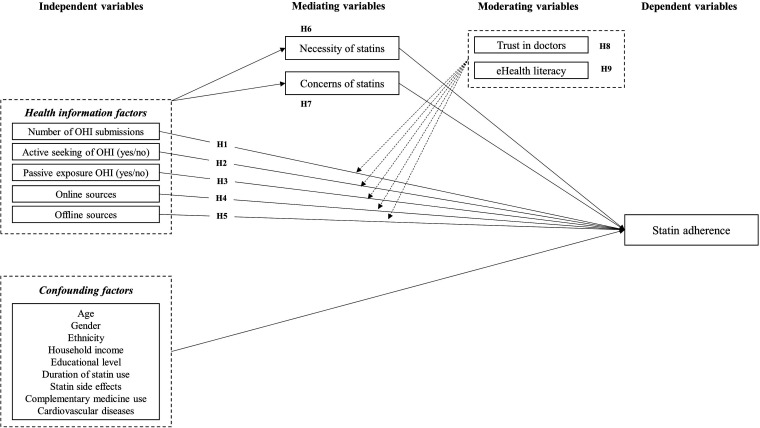
The structural model illustrates the hypothesised direct and indirect relationship between health information factors as independent factors, mediating variables, moderating variables and statin adherence as dependent variable. OHI, online health information; H, hypothesis.

A research model was developed based on a review of the literature and a qualitative study,^22^ which explored how online health information behaviour influences statin adherence. Statin adherence is the dependent variable and health information factors are the independent variables (Figure 1). Studies show that online health information is negatively associated with patients’ medication adherence,^26,27^ especially among those who use the Internet more frequently.^28^ The qualitative study shows that adherence to statins may be influenced differently depending on whether people are actively seeking or passively exposed to relevant health information, and that the specific source of information might also influence their decision to continue with statins.^22^

Based on what is already known, we propose the following hypotheses:

*H1:* Patients who more frequently encounter online health information are more likely to have a lower statin adherence
*H2:* Patients who actively seek online health information are more likely to have a lower statin adherence
*H3:* Patients who are passively exposed to online health information are more likely to have a lower statin adherence
*H4:* Patients who encounter online health information are more likely to have a lower statin adherence
*H5:* Patients who encounter offline health information are more likely to have a higher statin adherence

We also adapted the theoretical model proposed by Berglund et al.^19^ proposing that patient belief in the necessity of medications and concern about side effects are the main factors that influence medication adherence. Linn et al.^26^ found that patients who actively seek online health information may have more concerns about their medication than those who do not.

*H6:* Necessity of statins mediates the relationship between online health information behaviour and statin adherence
*H7:* Concern about statins mediates the relationship between online health information behaviour and statin adherence

Online health information behaviour has positive and negative impacts on patient-doctor relationships.^29^ Patients who trust in their doctors and discuss online health information with them will have less anxiety about the medication and exhibit higher levels of adherence.^30^ People with lower eHealth literacy may be more likely to trust low-quality online health information,^31^ and then more likely to be concerned about statin side effects (a common theme in low quality information about statins) and exhibit lower levels of adherence.^22^

*H8:* Trust in doctors moderates the relationship between online health information behaviour and statin adherence
*H9:* eHealth literacy moderates the relationship between online health information behaviour and statin adherence

Control variables of medication adherence are sociodemographic factors such as age, gender, ethnicity, household income and educational level; and health and treatment-related factors such as presence of cardiovascular disease, treatment duration, use of complementary medicines and experience of statin side effects.^19,32^

### Study design

This prospective cohort study used an information diary platform (IDP) to capture participant health information access and exposure prospectively for 2 months. The STROBE statement was used to guide the reporting.^33^

### Setting and participants

This study was conducted with patients who attended a primary care clinic at the University Malaya Medical Centre, Kuala Lumpur, Malaysia. We recruited patients who attended the primary care clinic between October 2022 and January 2023. Patients were included in the study if they met the following criteria: (a) aged 18 years or older; (b) patients have high cardiovascular risk where statin use was indicated as per Malaysian dyslipidemia management guideline,^34^ which are pre-existing CVD, diabetes mellitus, chronic kidney disease of stage 3 or higher, or a Framingham General CVD risk score of 20% or higher; and (c) patients who regularly use a smartphone. Pre-existing CVD, including coronary heart disease, stroke and peripheral vascular disease, was determined based on clinicians’ diagnosis and history of hospital admission for cardiovascular conditions from patients’ medical records. Patients were excluded if they were unable to read English or Malay, too ill during recruitment or cognitively impaired.

### Sample size

The sample size was calculated using OpenEpi sample size calculator for cohort study. We assumed a two-tailed coefficient alpha of 0.05 and a power of 80%. We estimated a sample size of 220 based on a longitudinal cohort by Linn et al.^26^ examining the mean difference in medication adherence between participants who sought information online compared to those who did not (0.55 ± 1.32 vs 0.17 ± 0.53). After accounting for a 20% loss to follow-up rate, the total number of participants to recruit at baseline was 264.

### Data collection process

**Figure 2.**
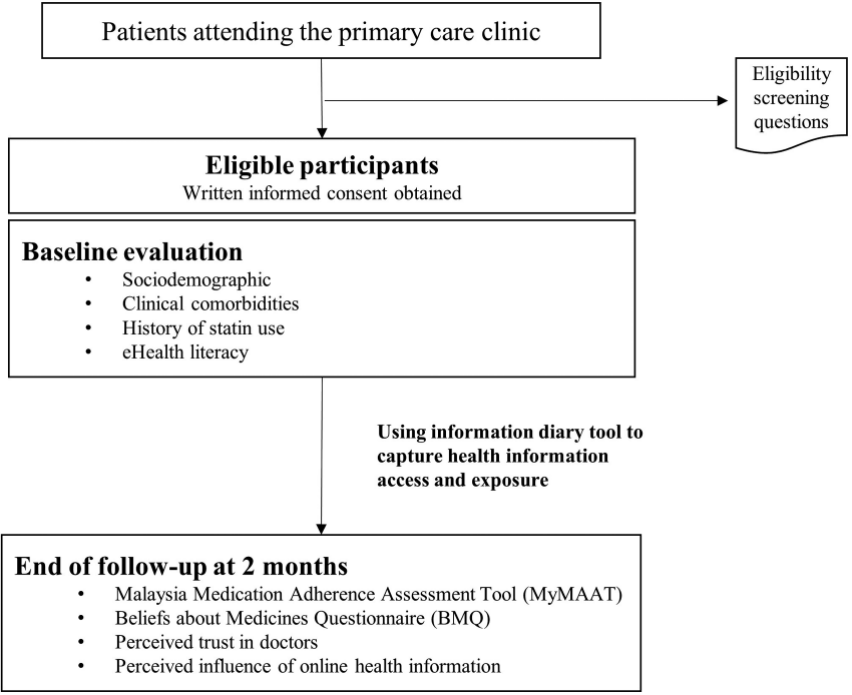
A schematic of the process of data collection shows evaluation at baseline and at the end of the observation period.

We searched the hospital electronic medical records to identify patients who fulfilled the inclusion criteria (Figure 2). Written consent was taken from eligible patients who agreed to participate in this study. The participants filled in the baseline questionnaire and installed the information diary tool application on their smartphones. The research team conducted a 30-minute training session with the participant to guide them on how to use the application. Participants were instructed to put an entry into the diary each time they encountered health information over a 2-month duration. At the end of 2 months, we contacted participants via phone call and sent them the questionnaire via email or WhatsApp message. Participants filled in an online questionnaire via REDCap.

### Study instruments

There were two study instruments used in this cohort study. The first instrument was the information diary tool (Appendix 1, Supplemental Figure S1), which was used to capture the health information encountered by the research participants for the 2-month observation period.^35^ The second instrument was a research questionnaire administered via the REDCap system following the conceptual framework (Appendix 1, Supplemental Table S1).

### Research instrument 1: information diary tool

An information diary tool was developed and tested to capture the health information that participants encountered either by actively searching or inadvertently while doing other things.^35,36^ In this study, participants were asked to use the information diary tool to record relevant health information they encountered for a period of 2 months. Health information in this study included information specific to statin use, information about lifestyle modification to improve cardiovascular health such as exercise and diet, information related to medications and complementary alternative medicine to improve cardiovascular health or treat dyslipidemia. Participants were asked to record health information they actively searched for (pull-based information) or health information that they encountered unintentionally, such as via advertisements, websites, or on social media (push-based information).

We defined a series of measures that aggregate the health information behaviour for each participant, with a focus on online health information (Table 1).

**Table 1.** Variables of online health information behaviour captured in information diary tool.

Variables	Types of variables	Operational definition
Number of online health information submissions	Continuous	Number of online health information submissions into the information diary tool in the past 2 months.
Active seeking of online health information	Dichotomous (Yes/No)	We categorised participants as ‘actively seeking for online health information’ when one or more of their submissions indicated that they specifically sought the information.
Passive exposure of online health information	Dichotomous (Yes/No)	We categorised participants as having ‘passive exposure of online health information’ when one or more of their submissions indicated that they did not specifically seek the information.
Number of submissions for each information source	Continuous	These included online sources such as Google, YouTube, Facebook, WhatsApp, Instagram, TikTok, and offline sources such as friends, family, and healthcare professionals. Participants were able to add any online or offline sources as free text beyond the existing set of options.

### Research instrument 2: questionnaire

Content validation was performed by an expert group of 3 senior lecturers in digital health, 2 family physicians, a professor in family medicine, and a pharmacist. The questionnaire was translated into Malay according to standard procedures.^37^ The questionnaire was pilot tested with 10 patients who had high cardiovascular risk.

There are six sections in this questionnaire. Section 1 captured the sociodemographic characteristics, including age, gender, ethnicity, educational level and household income. Section 2 was participants’ clinical characteristics, including presence of CVD (i.e. ischaemic heart disease, stroke and peripheral vascular disease), duration of statin use, experience of statin side effects and use of complementary alternative medicine. Section 3 measured participants’ eHealth literacy level using the eHealth Literacy Scale (eHEALS) instrument validated in both English and Malay languages in Malaysian populations.^38,39^ The eHEALS instrument evaluates participants’ perceived ability to find, evaluate and apply online health information. It consists of 8 items scored on a 5-point Likert scale ranging from 1 (strongly disagree) to 5 (strongly agree), with a total score ranging from 8 to 40 and a higher score indicating a better eHealth literacy level.

Section 4 measured statin adherence, the primary outcome of this study, using Malaysia Medication Adherence Assessment Tool (MyMAAT).^40^ MyMAAT was a 12-item questionnaire developed (English and Malay version) and validated in the Malaysian population to measure medication adherence. Four constructs measured medication-taking behaviour, perceived utility, perceived barriers, self-efficacy and social support. Individual items were scored using a 5-point Likert scale (1-strongly agree, to 5-strongly disagree). The total MyMAAT score ranges from 12 to 60; a score of 12–53 is categorised as non-adherence while a score of 54–60 is categorised as adherence. MyMAAT has a good internal consistency (Cronbach's alpha = 0.91) and excellent interclass correlation of 0.97.^40^

Section 5 was the Beliefs about Medicines Questionnaire (BMQ-Specific) to measure the necessity and concern of statins.^17^ Necessity items assessed participants’ beliefs of statin's necessity and perceived role of statins in CVD prevention. Concern items assessed participants’ beliefs on the danger and toxicity of statins. A five-point Likert scale (1 – strongly disagree, to 5 – strongly agree) is used for each item. A higher score indicates a stronger belief in the necessity of statins or a higher concern about statins. BMQ was validated in Malaysia with good Cronbach alpha of 0.86 and excellent interclass correlation of 0.92.^41^

Section 6 measured participants’ perception of trust in doctors and the influence of online health information on the decision to take statins. We also measured the perceived influence of online health information by asking participants whether health information they encountered on the Internet affected their decision to take statins using a 5-point Likert scale.

### Data analysis

Data were analysed using the Statistical Package for Social Sciences (SPSS) version 29 for descriptive statistical analyses and univariable inferential analyses. Continuous variables were tested for skewness and kurtosis via univariate normality testing. Independent T-tests were performed to compare the mean of concern and necessity scores between participants who were adherent and non-adherent to statins.

Structural equation modelling was conducted using partial least squares path modelling (PLS-SEM) to examine the extent to which our observed data fit with the a priori model proposed in the conceptual framework. PLS-SEM is a variance-based modelling approach to handle complex models with single and multi-item measures and analyse both formative and reflective models, and is well suited to exploratory research.^42^ SmartPLS version 4^43^ was used to construct the structural equation model for this study.

This study used four reflective constructs, i.e. necessity, concern, statin adherence and eHealth Literacy, and two formative constructs, i.e. online sources and offline sources (see Appendix 1 for the full description). For the structural model, path coefficient estimates (standardised regression coefficients, *β*) and their significance (*p*-value) were examined using a bootstrapping approach to examine the associations between independent, mediating, moderating and dependent variables. The coefficient of determination (*R*^2^) and effect size (*f*^2^) were used to measure the explanatory power of the path model, considering *p* < 0.05 as statistically significant.

## Results

### Study participants

**Figure 3.**
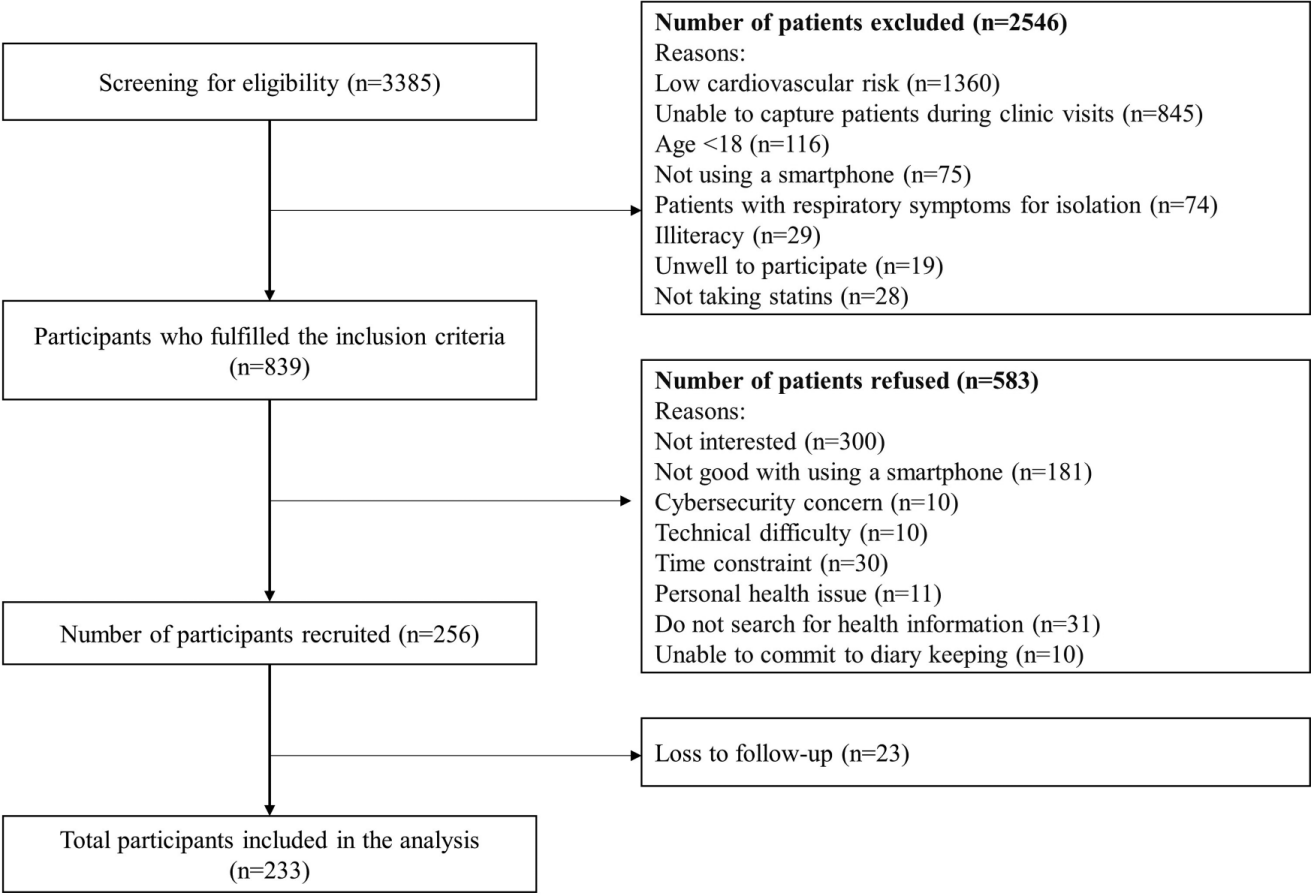
Flowchart of the recruitment of study participants.

A total of 256 patients were recruited in this study, which was 30.5% of the 839 who were approached and met the inclusion criteria (Figure 3). There was a 9.0% (*n* = 23) loss to follow-up after recruitment, and a total of 233 participants were included in the analysis. The mean age of participants was 59.82 ± 10.52 years, with 66.5% having an educational level of pre-university and above (Table 2). The mean eHealth literacy scale was 28.54 ± 5.27 (range 8–40). 26.2% of participants had pre-existing cardiovascular diseases and took statins for secondary prevention.

**Table 2.** Sociodemographic and clinical characteristics of study participants.

Variables	*N* (%)	Mean ± SD
**Sociodemographic characteristics**		
Age (years)		59.82 ± 10.52
Gender		
Male	122 (52.4)	
Female	111 (47.6)	
Ethnicity		
Malay	99 (42.5)	
Chinese	88 (37.8)	
Indian	43 (18.5)	
Others	3 (1.2)	
Educational level		
Primary education	6 (2.6)	
Lower secondary education	15 (6.4)	
Upper secondary education	57 (24.5)	
Pre-university	60 (25.8)	
Tertiary education	95 (40.7)	
Household income		
<RM3000	91 (39.0)	
RM3000–6000	81 (34.8)	
>RM6000	61 (26.2)	
eHealth literacy scale		28.54 ± 5.27
**Clinical characteristics**		
Existing cardiovascular diseases	61 (26.2)	
Duration of statin use (years)		6.48 ± 6.23 (range 1–40)
Experienced statin side effects	44 (19.0)	
Use of complementary alternative medicine in the past 2 months	24 (10.3)	

SD, standard deviation.

### Online health information behaviour

Data from the information diary tool showed that 62.7% (*n* = 146) of participants encountered health information from either online or offline sources in the 2-month study period; and 55.8% (*n* = 130) of the participants encountered online health information either through searching or through a passive encounter. The participants recorded a total of 978 online health information submissions, and the number of recorded submissions per participant ranged from 1 to 109 (median 1, IQR 5). Online health information was actively searched for by 39.1% (*n* = 91) of participants, 40.3% (*n* = 94) reported passive exposure to online health information, and 24.5% (*n* = 57) reported both actively seeking of and passive exposure to online health information. Participants reported Google (66.2%), YouTube (27.7%) and Facebook (27.7%) as the three main sources of online health information submissions (Table 3). There was a total of 197 submissions related to offline health information, including from healthcare providers (16.2%), friends (14.6%), family (11.5%) and newspapers (6.2%).

**Table 3.** Descriptive analysis of health information behaviour from the information diary tool (*N* = 146).

Number of submissions in the diary tool	Proportion of participants (*n*, %)	Range of submissions
Online sources		
Google	86 (58.9)	1–72
YouTube	36 (24.7)	1–22
Facebook	36 (24.7)	1–106
WhatsApp	22 (15.1)	1–4
Instagram	4 (2.7)	1–2
TikTok	10 (6.8)	2–20
Offline sources		
Healthcare professionals	21 (14.4)	1–7
Friends	19 (13.0)	1–19
Family	15 (10.3)	1–5
Newspapers	8 (5.5)	1–31

Results from the questionnaire survey tool showed that only 19.7% believed that online health information affected their decision whether or not to take statins (Table 4). Conversely, 90.1% of participants trusted health information from their doctors. Among 130 participants who submitted online health information in the information diary tool, only 42.3% (*n* = 55) reported discussing online health information with their doctors.

**Table 4.** Descriptive analysis of online health information behaviour from the questionnaire tool.

Items from the questionnaire tool	*N* (%)
*Among participants recruited in this study (n = 233),*	
The health information I encountered on the Internet affected my decision to take statins	
Strongly agree	7 (3.0)
Agree	39 (16.7)
Unsure	37 (15.9)
Disagree	76 (32.6)
Strongly disagree	74 (31.8)
I trust health information from my doctor	
Strongly agree	71 (30.4)
Agree	139 (59.7)
Unsure	16 (6.9)
Disagree	2 (0.9)
Strongly disagree	5 (2.1)
*Among participants who had online health information submissions in the information diary tool(n = 130),*	
Have discussed online health information with doctors	55 (42.3)
Reasons of not discussing with doctors	
Doctors were too busy	19 (14.6)
Doctors were not interested in online health information	7 (5.4)
The online health information is about complementary alternative medicine	23 (17.7)
The online health information is irrelevant to doctors	22 (16.9)

### Beliefs in statins and statin adherence

Among the 233 participants analysed, 52.4% (*n* = 122) did not adhere to statins as recommended or prescribed (MyMAAT score <54). Participants who were non-adherent had a higher concern score compared to participants who were adherent to statins (*T*-test, 17.19 vs 14.59, *p* < 0.001) (Appendix 2, Supplemental Table S1). There was no evidence of an association between necessity and adherence (15.60 vs 16.08, *p* = 0.358).

### Structural equation modelling

For reflective model assessment, all four reflective constructs (i.e. necessity, concern, statin adherence and eHealth literacy), had good internal consistency and convergent validity (Appendix 2, Supplemental Table S2). Discriminant validity assessment of all latent constructs showed good discriminant validity (Appendix 2, Supplemental Tables S3 and S4). There was no significant multicollinearity among the indicators (Appendix 2, Supplemental Table S5).

For the formative model assessment, online and offline sources were considered as formative constructs. Using outer loading and outer weight, only YouTube was included as an indicator for online source while friends, family and healthcare providers were included as indicators for offline sources (Appendix 2, Supplemental Table S6).

**Figure 4.**
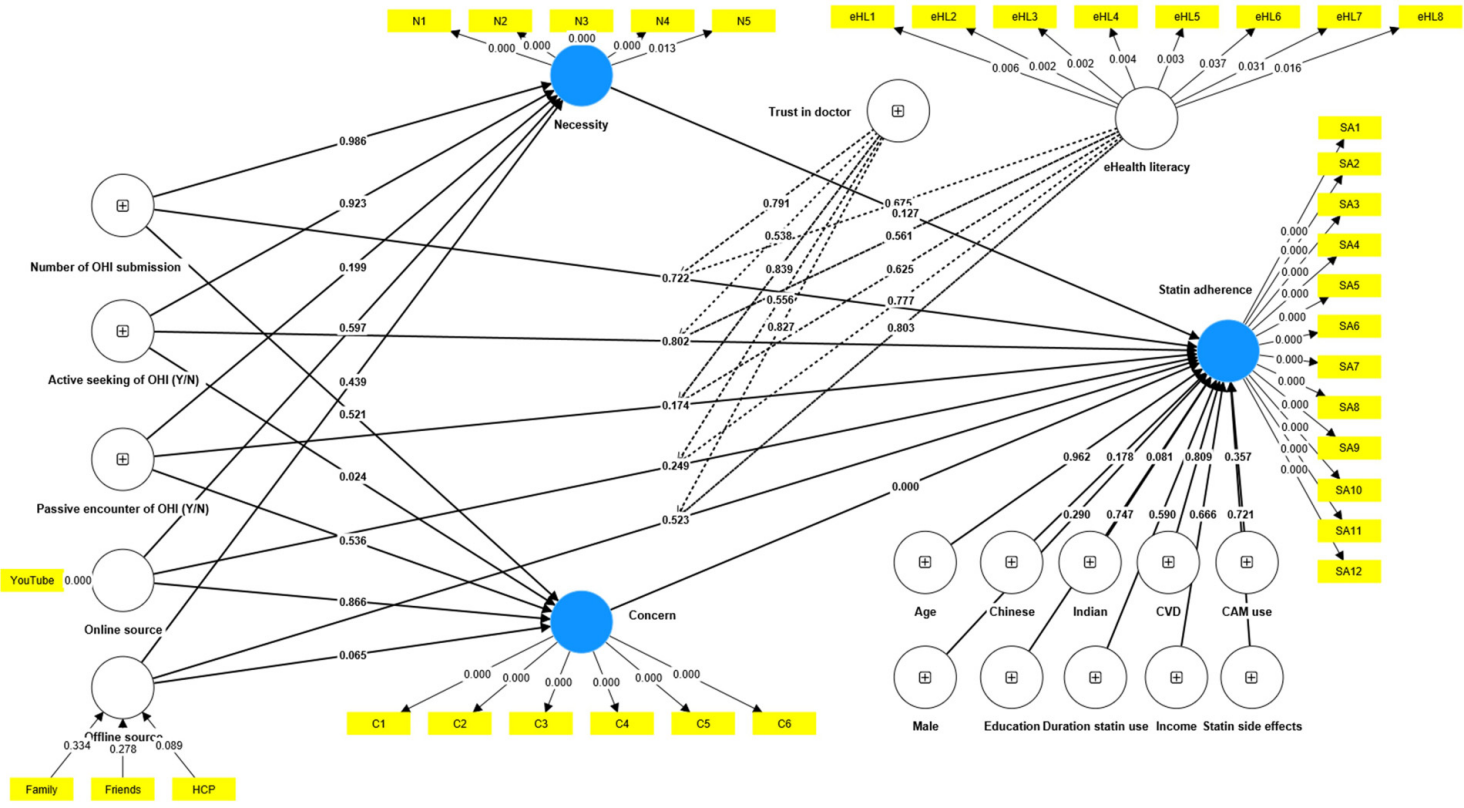
Structural equation modelling analysis that includes confounding factors.

After fitting the measurement model, we analysed the structural model to identify the associations between the independent variables and statin adherence (dependent variable) according to our a priori model (Figure 4). We examined the associations between confounding factors and statin adherence (Appendix 2, Supplemental Table S7). Participant ethnicity (reporting Chinese or Indian ethnicity) produced a *p*-value <0.25, and these factors were included in the structural model analysis.

OHI, online health information; CVD cardiovascular disease; CAM, complementary alternative medicine.

**Figure 5.**
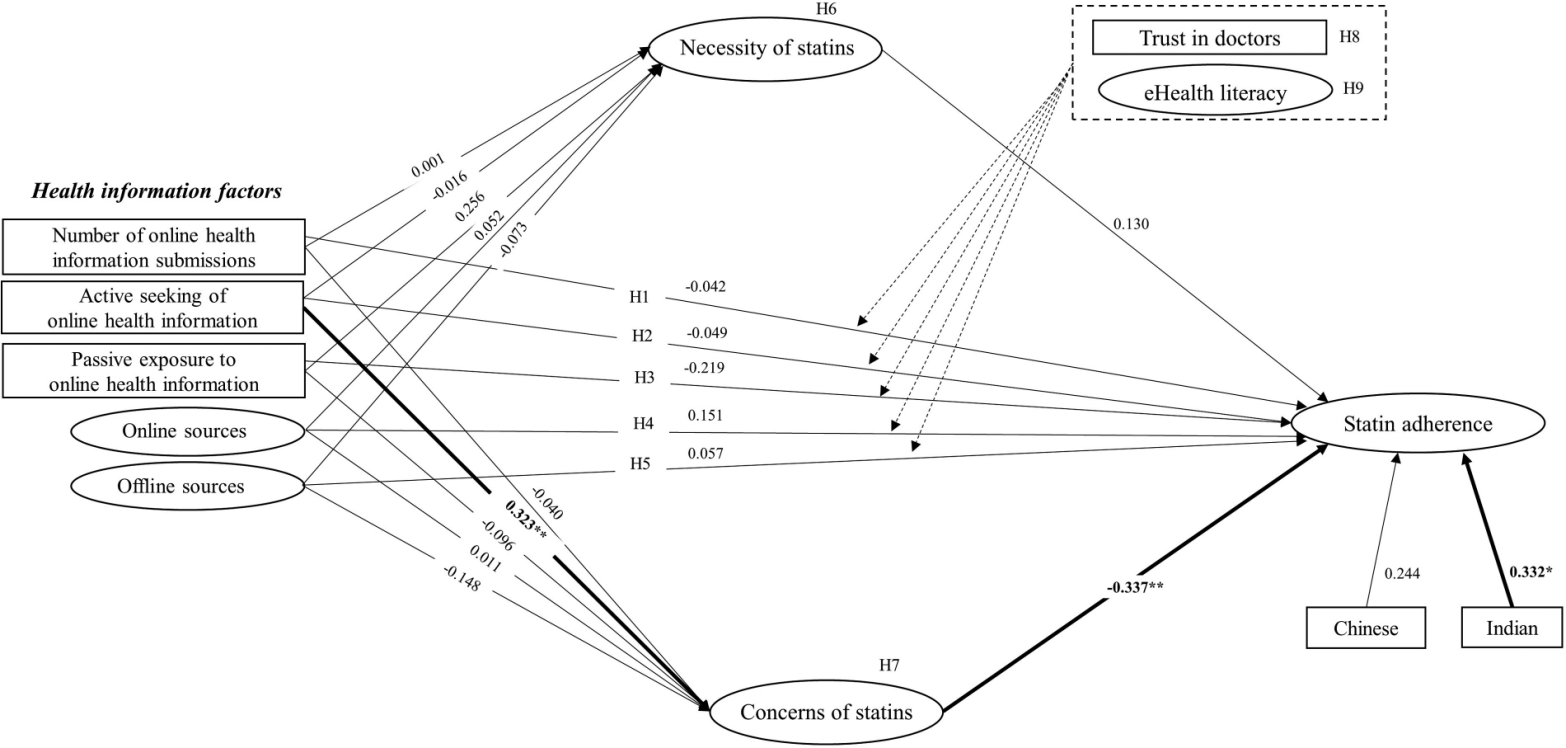
Structural equation modelling analysis with standardised path coefficients (*β*).

In the final structural model (Figure 5), the results showed that participants who actively sought online health information reported higher levels of concern about statin use (*β* = 0.323, *p* = 0.023). Participants with higher levels of concern about statin use were less likely to adhere to statins (*β* = −0.337, *p* < 0.001). Participants reporting Indian ethnicity also reported higher levels of statin adherence (*β* = 0.332, *p* = 0.050). Sources of information encountered by participants (whether online or offline) were not associated with concern, necessity, or statin adherence (see full details of the path analysis in Appendix 2, Supplemental Table S8). For the explanatory power assessment, *R*^2^ for independent variables to statin adherence was 0.251, indicating a satisfactory predictive power. The *R*^2^ for concern (0.042) and necessity (0.023) showed poor predictive power (Appendix 2, Supplemental Tables S9 and S10).

Bold lines indicate significant relationships. *p < 0.05, **p < 0.001. Latent constructs are ovals, single-item variables are rectangles.

For the mediation analysis, concern about statins significantly mediated the relationship between active-seeking of online health information and statin adherence (indirect effect, *β* = -0.109, *p* = 0.048). The results showed that the indirect effect (active-seeking of online health information → concern → statin adherence) was significant (*p* = 0.048), but the direct effect (active-seeking of online health information → statin adherence) was not significant (*p* = 0.739), indicating that concern about statins fully mediated the relationship between active-seeking of online health information and statin adherence. This means that we found evidence that the active seeking of online health information was associated with a lower statin adherence (total effect *β* = −0.160), and this relationship was mediated by concern about statins. Test of indirect effects for other health information variables produced no significant results (Appendix 2, Supplemental Table S11).

For moderation analysis, our findings showed that trust in doctors and higher levels of eHealth literacy did not strengthen or weaken the relationship between online health information seeking behaviour and statin adherence (Appendix 2, Supplemental Table S12).

### Subgroup analysis

We sought to further examine differences between participants who actively sought online health information and those who did not (Table 5). An independent T-test was performed to compare characteristics between participants who reported active-seeking of online health information (n = 91) and those who did not (i.e. participants who reported passive online health information exposure only and those who did not encounter any online health information, *n *= 142).

**Table 5.** Comparison between participants who reported active online health information-seeking and those who did not.

	Participants reported active OHI-seeking (*n* = 91)	Participants who did not report active OHI-seeking^a^ (*n* = 142)	*P*-value
Age (mean ± SD)	58.77 ± 10.3	60.50 ± 10.64	0.221
Duration of statin use, years (mean ± SD)	5.85 ± 5.69	6.88 ± 6.54	0.219
eHealth literacy scale (mean ± SD)	29.96 ± 3.86	27.63 ± 5.84	<0.001
Perceived influence of OHI on decisions to take statins (mean ± SD)	2.49 ± 1.16	2.12 ± 1.15	0.016
Trust in doctors (mean ± SD)	4.07 ± 0.83	4.21 ± 0.71	0.156
Male (*n*, %)	49 (53.8)	73 (51.4)	0.716
Ethnicity (*n*, %)			0.105
Malay	40 (44)	59 (41.5)	
Chinese	40 (44)	48 (33.8)	
Indian	10 (11)	33 (23.2)	
Experienced statin side effects (*n*, %)	15 (16.5)	29 (20.4)	0.454
Existing cardiovascular diseases (*n*, %)	18 (19.8)	43 (30.3)	0.075
Use of complementary alternative medicine (*n*, %)	8 (8.8)	16 (11.3)	0.544

OHI, online health information; SD, standard deviation.

^a^ Participants who did not report active online health information-seeking were those who reported passive online health information exposure and those who did not seek online health information.

The results of this subgroup analysis showed that people who actively sought online health information reported a higher eHealth literacy scale (eHEALS score 29.96 vs 27.63, *p* < 0.001). Participants who actively sought online health information were also more likely to report that online health information influenced their decisions on whether to take statins compared to those who did not seek online health information (2.49 vs 2.12, *p* = 0.016). We did not find evidence that participants who actively sought online health information had a lower trust in doctors compared to participants who did not seek online health information (4.07 vs 4.21, *p* = 0.156).

## Discussion

We investigated the relationships between online health information behaviour and statin adherence. To our knowledge, this was the first study to measure health information behaviour in detail using an information diary app. The results of the study showed that patients who actively sought online health information had higher concerns about statin use and lower levels of statin adherence. While we found that concern about statins was associated with lower statin adherence, we did not find evidence of an association between perceived necessity of statins and statin adherence. Among the confounding factors, only ethnicity was found to have a direct association with statin adherence. Trust in doctors and eHealth literacy were not found to be moderating factors in the relationship between online health information behaviour and statin adherence.

Other studies have examined the topic at different levels of granularity. Linn et al.^26^ reported that patients who sought online health information during the treatment had higher concerns about the medication than those who did not seek online health information. Where the main sources of health information are Google, YouTube and Facebook, this may reflect the dominance of content about side effects or the specific information that people with concerns search for.^44^ Our subgroup analysis showed that patients who actively sought online health information were also more likely to report that online health information influenced their decision to take statins. When patients perceive health information as useful and important, it is more likely to result in behavioural change.^45^ Our findings are aligned with previous studies of cardiovascular health showing that exposure to online health information that exaggerates statin side effects is associated with statin non-adherence.^46,47^ Other related studies have suggested that online searches may contribute to a nocebo effect, where patients who expect side effects are more likely to experience them.^48^

Our results contrast with a previous study that showed necessity of statins is more closely associated with statin adherence than concern in patients with hypercholesterolemia.^19^ The discrepancy could be explained by the longer duration of statin use (median 6 years, range 1–40 years) in our study population, with the majority (73.8%) taking statins as primary prevention. As patients with hypercholesterolemia were asymptomatic where statin use could not provide symptom relief or noticeable efficacy, necessity of medication becomes less influential on statin adherence.^20^

We did not find evidence that trust in doctors had a moderating influence on the relationship between online health information behaviour and statin adherence. A systematic review found that patient-doctor relationship is an important factor in moderating the influence of online health information behaviour on health outcomes.^11^ When patients encounter good-quality information that is concordant with advice from doctors, it increases their trust in doctors and subsequently enhances their treatment adherence.^49^ Good communication between patients and doctors to align their agreement regarding medical treatment can counteract the negative influence of poor-quality online information.^50^ When doctors offer patients with additional online health information sources to reinforce their recommendations or prescriptions, doctors need to assess the patient's capacity to comprehend, critically evaluate and apply the online health information. For patients with limited health literacy, it is the responsibility of doctors to proactively engage them and provide patient education materials with appropriate readability.^51^

We found no evidence that eHealth literacy moderated the relationship between health information behaviour and statin adherence. Lu et al.^52^ concluded that eHealth literacy improved patient appraisal of information quality and improved treatment adherence. Our sub-group analysis showed that patients who actively sought online health information had a higher eHealth literacy compared to participants who did not search for online health information. It is possible that patients with high eHealth literacy may be better at searching for information online but may not appraise the quality of health information or use the appraisal of credibility in their decision making.^38,53^

For sociodemographic factors, patients with reported Indian ethnicity had a higher statin adherence in our study. This result was consistent with previous research conducted in Malaysia where Indian patients often reported to have a higher medication adherence for chronic diseases.^54^ However, other studies conducted in Malaysia did not show any clear association between ethnicity and medication adherence in patients with high cardiovascular risks.^55,56^ The differences in statin adherence among ethnic groups could be related to their socio-cultural background, and their beliefs and perceptions about medical treatments.^57^

### Research and practice implications

This study has implications for development of research in online health information behaviour because it examined the ‘real world’ behaviours of statin adherence in much greater detail than was possible in previous studies that measured health information behaviour in different ways. We made use of a health information model that delineates between active and passive health information behaviours^58^; and augmented the model by comparing different online health information behaviours relative to medication adherence. The integrative theoretical framework used in the design of this study may be useful for studying online health information behaviour in relation to other conditions where people make choices, such as vaccination.

The findings also have implications for practice. The results suggest that doctors may improve adherence in their patients by focusing on concerns about statin use in their consultations and consider discussing common misconceptions that can be found online. We suggest that doctors could actively initiate discussions with patients about their online health information behaviour, identify patients who might be influenced by online misinformation, and provide patients with lists of high quality and evidence-based health information. The results also suggest that doctors should not assume that patients with higher educational levels are at a lower risk of being influenced by lower quality online health information.

### Limitations and future research

This study has limitations. First, the participants recruited in this study had relatively high levels of education and may not represent populations in other primary care settings. Second, a 2-month observation period only captured a snapshot of health information behaviour which does not allow causal inferences. A larger longitudinal cohort study with a longer duration may produce a more comprehensive view of online health information behaviour. Third, noncompliance with a diary method in data collection is common and unavoidable. We were unable to ascertain their compliance with recording health information in the diary tool. Future research could consider improvements and automation in the research instruments used to capture online health information behaviour by considering passive data acquisition of health information exposure using technology.^36^ Fourth, we did not critically appraise the quality of the online health information that participants encountered, but this may be a useful way to better understand differences in the quality of the sources of information. Fifth, our proposed model has a low explanatory power, which may be due to the sample size and the number of variables included in the model. Future studies in the area may consider the use of explainable machine learning methods to identify factors that are important in predictive models of medication adherence that include health information sources, conduits, and patient demographics. We suggest that additional information about medication adherence from before the start of the observation period might be useful to consider in future studies. Future studies in this area may consider examining how online health information might influence the dynamic of traditional doctor-patient relationships when patients were empowered and have the access to online health information.

## Conclusion

The influence of online health information behaviour on medication adherence is complex. We found that patients with high cardiovascular risk who actively searched for online health information had lower statin adherence, and this relationship was mediated by higher concerns about statins. The strengths of the study included studying an appropriate population and the breadth and detail with which we recorded the health information that people accessed. The results suggest that when health professionals talk to patients about statins, they may want to directly address concerns and provide evidence-based information.

## Supplemental Material

sj-docx-1-dhj-10.1177_20552076241241250 - Supplemental material for Online health information behaviour and its association with statin adherence in patients with high cardiovascular risk: A prospective cohort study Supplemental material, sj-docx-1-dhj-10.1177_20552076241241250 for Online health information behaviour and its association with statin adherence in patients with high cardiovascular risk: A prospective cohort study by Hooi Min Lim, Chirk Jenn Ng, Adina Abdullah, Mahmoud Danee, Jacques Raubenheimer and Adam G. Dunn in DIGITAL HEALTH

sj-docx-2-dhj-10.1177_20552076241241250 - Supplemental material for Online health information behaviour and its association with statin adherence in patients with high cardiovascular risk: A prospective cohort study Supplemental material, sj-docx-2-dhj-10.1177_20552076241241250 for Online health information behaviour and its association with statin adherence in patients with high cardiovascular risk: A prospective cohort study by Hooi Min Lim, Chirk Jenn Ng, Adina Abdullah, Mahmoud Danee, Jacques Raubenheimer and Adam G. Dunn in DIGITAL HEALTH

## Abbreviations

CVD: cardiovascular diseases
NCF: Necessity-Concern Framework
IDP: Information diary platform
OHI: online health information
eHEALS: eHealth literacy scale
BMQ: Beliefs about Medicines Questionnaire
