# Supplementary material for: Online health information behaviour and its association with statin adherence in patients with high cardiovascular risk: A prospective cohort study
Source: Digit Health. 2024 Mar 21;10:20552076241241250. doi: 10.1177/20552076241241250 (PMC10956144; doi:10.1177/20552076241241250)
Supplement: sj-docx-1-dhj-10.1177_20552076241241250 - Supplemental material for Online health information behaviour and its association with statin adherence in patients with high cardiovascular risk: A prospective cohort study [file sj-docx-1-dhj-10.1177_20552076241241250.docx]

**Appendix 1**

This cohort study used an information diary tool (Figure S1) to capture health information encountered by research participants for 2 months. They chose the category and source of health information they encountered. Participants indicated whether they specifically searched for the information. It is optional to describe the content, copy the link of online information or attach an image for offline information. Within the health information recorded by participants in the diary tool, 23% of entries include an image, 75% contain a link and 45% describe the information content.

| 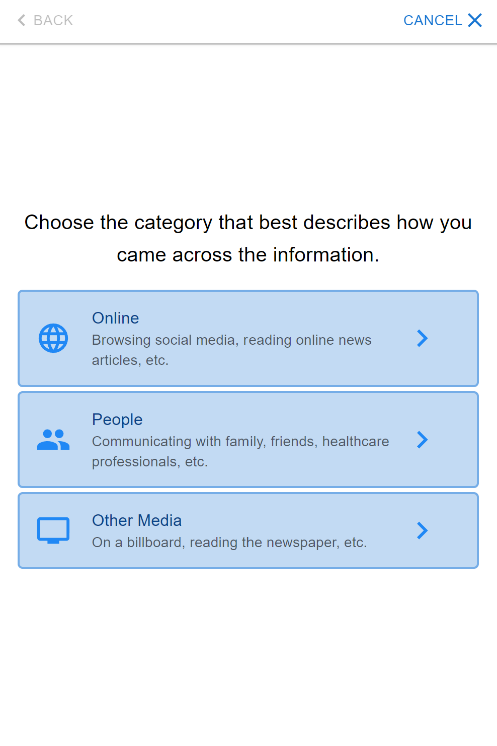 | 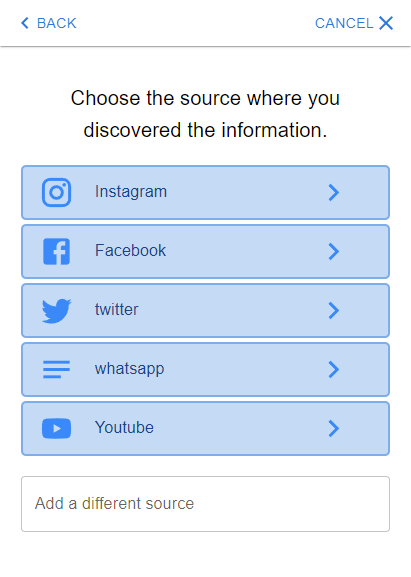 | 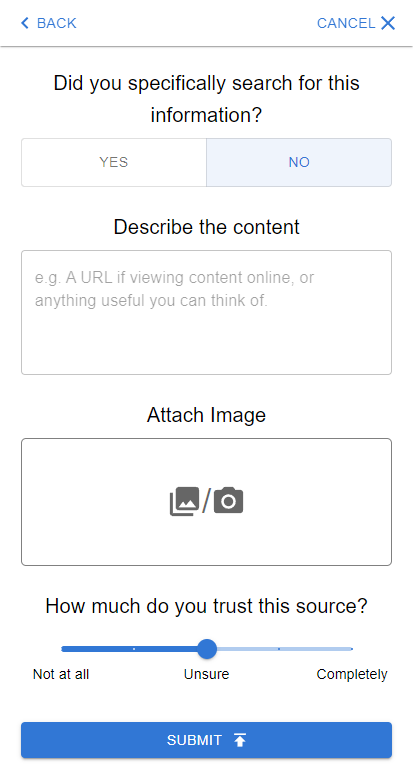 |
| --- | --- | --- |

**Figure S1. Information diary tool**

**Table S1. Quantitative survey items and scales development based on themes and subthemes from qualitative data analysis**

| **Themes and subthemes from the qualitative study (Lim et al, 2023)** | **Corresponding survey items/variables in the quantitative cohort study** |
| --- | --- |
| Theme: Seeking OHI throughout the disease trajectory   - When doctors started statins - When cholesterol levels were uncontrolled, and doctors changed medications - When cholesterol levels were under control | *Item*: Duration of statin use (years) |
| Theme: Active and passive approaches to seeking OHI   - Being pushed with information - Random search via search engines - Targeted online sources - Followed influencers on social media | *IDP*: Participants specify whether they actively search for the OHI for each submission  *IDP*: Sources online information encountered by participants; sources of offline information |
| Theme: Views about statin-related OHI   - Frame negatively as side effects - Perceived as a pharmaceutical tactic | *Questionnaire*: BMQ Concerns of medicine |
| Theme: Influence of OHI on patients’ health decisions   - Reassured on statins use - Hesitated to continue statins - Triggered participants to look for alternative treatment - Confused with conflicting information - Triggered negative emotions | *Item*: The health information I encountered on the internet affected my decision to take statins (5-point Rating scale)  *Item*: Use of complementary alternative treatment  *Questionnaire:* eHealth literacy scale |
| Theme: Patient-doctor communication about OHI   - Trust in doctors - Perceived doctors were busy and not interested in OHI - Perceived OHI was irrelevant to doctors - Doctors’ response towards patients’ OHI-seeking behaviour | *Item*: I trust health information from my doctors (5-point Rating scale)  *Item*: Have you ever discussed OHI with your doctor? If not, what are the reasons for not discussing this with your doctor? |

IDP, Information diary platform; OHI, online health information; BMQ, Beliefs in Medicines Questionnaire; MyMAAT, Malaysia Medication Adherence Assessment Tool

Lim HM, Ng CJ, Dunn AG, Abdullah A (2023). Experiences and influences of online health information-seeking in patients with high cardiovascular risks: a qualitative study. *Family Practice*, cmad034. [doi.org/10.1093/fampra/cmad034](https://doi.org/10.1093/fampra/cmad034)

**Data analysis of PLS-SEM**

**Reflective and formative constructs**

In this study, there are 4 reflective constructs, i.e. necessity and concern of statins, statin adherence and eHealth literacy. A reflective construct refers to a latent variable where the indicators are caused by the construct and indicators with the same construct are highly correlated with each other. In contrast, a formative construct refers to a composite variable where indicators cause the construct and each indicator captures a specific aspect of a construct’s domain. The indicators of a formative construct are not correlated or interchangeable. In this study, there are 2 formative constructs which are online sources and offline resources.

**Reflective model assessment**

For reflective measurement model, we assessed the construct reliability, convergent validity and discriminant validity. For construct reliability, we tested the model using Cronbach’ coefficient (α) reliability, composite reliability rho_a and composite reliability rho_c (≥ 0.70 indicates internal consistency reliability) [1]. Convergent validity was assessed using average variance extracted (AVE) where an AVE larger than 0.5 shows good validity. We also assessed the outer loadings of items where a loading of ≥ 0.70 indicates convergent validity. An item with a loading of <0.40 should be removed from the construct while an item with a loading between 0.40 and 0.70 can be removed if the removal improves the composite reliability and AVE [1]. Discriminant validity was assessed using three methods: Fornell-Larcker criterion, Heterotrait-monotrait (HTMT) criterion and cross-loadings. For the Fornell-Larcker criterion, convergent validity is achieved when the square root of AVE of a latent variable is larger than its correlations with other latent variables [1]. For HTMT criterion, a HTMT value of <0.85 indicates good discriminant validity. For cross-loadings method, convergent validity is achieved when the loading value of an indicator to its latent variable is larger than its cross-loading value in other latent variables [1]. Also, we assessed the presence of multicollinearity among constructs using variance inflation factor (VIF).

**Formative model assessment**

For formative measurement model, we assessed the collinearity, significance and relevance of the indicator weights. There were two formative constructs in this study, i.e., Online Source and Offline Source. We analysed the VIF of each indicator to determine the collinearity between formative indicators (VIF < 5 indicates no collinearity issues). To assess the significance and relevance of indicator weights, we used the outer weight to determine the contribution of an indicator to a formative construct. An indicator would be retained when an indicator’s outer weight is significant. When the outer weight is non-significant, but its outer loading is high (>0.50), an indicator could be kept too. An indicator would be removed when the outer weight is non-significant and the outer loading is <0.50 [1].

References:

1. Hair, J., et al., *A Primer on Partial Least Squares Structural Equation Modeling (PLS-SEM)*. 2022. Sage: Los Angeles.

2. Jr, H., et al., *When to use and how to report the results of PLS-SEM.* European Business Review, 2018. **31**.

3. Benitez, J., et al., *How to perform and report an impactful analysis using partial least squares: Guidelines for confirmatory and explanatory IS research.* Information & Management, 2020. **57**(2): p. 103168.
