# Supplementary material for: Online health information behaviour and its association with statin adherence in patients with high cardiovascular risk: A prospective cohort study
Source: Digit Health. 2024 Mar 21;10:20552076241241250. doi: 10.1177/20552076241241250 (PMC10956144; doi:10.1177/20552076241241250)
Supplement: sj-docx-2-dhj-10.1177_20552076241241250 - Supplemental material for Online health information behaviour and its association with statin adherence in patients with high cardiovascular risk: A prospective cohort study [file sj-docx-2-dhj-10.1177_20552076241241250.docx]

**Appendix 2**

**Beliefs in statins and statins adherence**

**Table S1. Difference of Concern and Necessity of statins between adherent and non-adherent participants**

|  | **Non-adherence** (n=122) | **Adherence**  (n=111) | **Mean difference**  **(95% CI)** | **p-value** |
| --- | --- | --- | --- | --- |
|  | Mean ± SD | Mean ± SD |  |  |
| Concern | 17.19 ± 3.92 | 14.59 ± 4.76 | 2.594 (1.472-3.716) | <0.001 |
| Necessity | 15.60 ± 3.45 | 16.08 ± 4.52 | 0.358 (-1.516-0.550) | 0.358 |

**Reflective model assessment**

All four reflective constructs in our study, i.e. Necessity, Concern, Statin Adherence and eHealth Literacy (Table S2), have a Cronbach’s alpha ranging from 0.792-0.902 (>0.70), a composite reliability rho_a ranged from 0.799-0.969 (>0.70) and a composite reliability rho_c ranged from 0.850-0.931 (>0.70). All constructs showed good convergent validity (AVE >0.5), except Concern (AVE= 0.488), however, it is very near to 0.50.

**Table S2. Internal consistency and convergent validity of reflective constructs**

| **Construct** | **Indicator** | **Outer Loading** | **Cronbach’s α** | **Composite reliability (rho_a)** | **Composite reliability (rho_c)** | **Average Variance Extracted (AVE)** |
| --- | --- | --- | --- | --- | --- | --- |
| Concern | C1 | 0.680 | 0.791 | 0.802 | 0.850 | 0.487 |
|  | C2 | 0.663 |  |  |  |  |
|  | C3 | 0.614 |  |  |  |  |
|  | C4 | 0.769 |  |  |  |  |
|  | C5 | 0.742 |  |  |  |  |
|  | C6 | 0.705 |  |  |  |  |
| Necessity | N1 | 0.680 | 0.804 | 0.823 | 0.857 | 0.546 |
|  | N2 | 0.805 |  |  |  |  |
|  | N3 | 0.791 |  |  |  |  |
|  | N4 | 0.737 |  |  |  |  |
|  | N5 | 0.672 |  |  |  |  |
|  |  |  |  |  |  |  |
| Statin adherence | SA1 | 0.677 | 0.919 | 0.927 | 0.931 | 0.531 |
|  | SA2 | 0.805 |  |  |  |  |
|  | SA3 | 0.777 |  |  |  |  |
|  | SA4 | 0.795 |  |  |  |  |
|  | SA5 | 0.770 |  |  |  |  |
|  | SA6 | 0.671 |  |  |  |  |
|  | SA7 | 0.555 |  |  |  |  |
|  | SA8 | 0.660 |  |  |  |  |
|  | SA9 | 0.714 |  |  |  |  |
|  | SA10 | 0.736 |  |  |  |  |
|  | SA11 | 0.817 |  |  |  |  |
|  | SA12 | 0.722 |  |  |  |  |
| eHealth Literacy | eHL1 | 0.802 | 0.904 | 0.993 | 0.921 | 0.599 |
|  | eHL2 | 0.893 |  |  |  |  |
|  | eHL3 | 0.910 |  |  |  |  |
|  | eHL4 | 0.843 |  |  |  |  |
|  | eHL5 | 0.829 |  |  |  |  |
|  | eHL6 | 0.599 |  |  |  |  |
|  | eHL7 | 0.618 |  |  |  |  |
|  | eHL8 | 0.621 |  |  |  |  |

**Discriminant validity**

For Fornell-Larcker criterion (Table S3), the square root of the AVE value for Concern (0.697), Necessity (0.739), Statin adherence (0.728) and eHealth literacy (0.774) were larger than their correlations with other measurement models. HTMT values of all the pairs of the latent variables were <0.90.

**Table S3. Fornell-Larcker and Heterotrait-monotrait criteria for discriminant validity assessment**

| **Fornell-Larcker criterion** | **Concern** | **Necessity** | **Statin adherence** | **eHealth literacy** |
| --- | --- | --- | --- | --- |
| Concern | **0.697** |  |  |  |
| Necessity | 0.038 | **0.739** |  |  |
| Statin adherence | -0.390 | 0.115 | **0.728** |  |
| eHealth literacy | 0.059 | 0.076 | 0.054 | **0.774** |
| **Heterotrait-monotrait criterion** |  |  |  |  |
| Concern |  |  |  |  |
| Necessity | 0.232 |  |  |  |
| Statin adherence | 0.426 | 0.146 |  |  |
| eHealth literacy | 0.123 | 0.104 | 0.088 |  |

C: Concern; N, Necessity; SA: Statin adherence, eHL: eHealth literacy

For cross-loadings (Table S4), results showed that the cross-loading values of the indicators of each of the constructs were larger than their cross-loading values with other constructs.

**Table S4. Cross-loadings of constructs for discriminant validity assessment**

|  | **Concern** | **Necessity** | **Statin adherence** | **eHealth literacy** |
| --- | --- | --- | --- | --- |
| C1 | **0.680** | -0.028 | -0.224 | 0.025 |
| C2 | **0.663** | 0.058 | -0.170 | 0.117 |
| C3 | **0.614** | 0.033 | -0.222 | -0.044 |
| C4 | **0.770** | 0.040 | -0.361 | 0.026 |
| C5 | **0.742** | 0.143 | -0.285 | 0.092 |
| C6 | **0.704** | -0.071 | -0.307 | 0.051 |
| N1 | 0.044 | **0.679** | 0.088 | 0.075 |
| N2 | 0.088 | **0.804** | 0.060 | 0.085 |
| N3 | 0.193 | **0.790** | 0.004 | 0.085 |
| N4 | 0.138 | **0.736** | 0.012 | 0.019 |
| N5 | -0.182 | **0.673** | 0.186 | 0.024 |
| SA1 | -0.351 | 0.150 | **0.781** | 0.029 |
| SA2 | -0.379 | 0.090 | **0.791** | 0.039 |
| SA3 | -0.294 | 0.054 | **0.765** | 0.140 |
| SA4 | -0.302 | 0.033 | **0.679** | -0.034 |
| SA5 | -0.231 | 0.100 | **0.55** | -0.061 |
| SA6 | -0.305 | 0.143 | **0.812** | 0.046 |
| SA7 | -0.151 | 0.074 | **0.685** | 0.042 |
| SA8 | -0.319 | 0.172 | **0.799** | 0.015 |
| SA9 | -0.284 | 0.027 | **0.664** | 0.072 |
| SA10 | -0.269 | 0.032 | **0.713** | 0.055 |
| SA11 | -0.197 | 0.072 | **0.736** | 0.053 |
| SA12 | -0.221 | 0.008 | **0.726** | 0.058 |
| eHL1 | 0.047 | 0.080 | 0.020 | **0.806** |
| eHL2 | 0.083 | 0.062 | 0.070 | **0.895** |
| eHL3 | 0.086 | 0.068 | 0.043 | **0.912** |
| eHL4 | 0.044 | 0.103 | 0.042 | **0.846** |
| eHL5 | 0.103 | 0.016 | 0.020 | **0.831** |
| eHL6 | -0.045 | 0.057 | 0.035 | **0.593** |
| eHL7 | -0.021 | 0.003 | 0.030 | **0.610** |
| eHL8 | 0.064 | 0.075 | 0.010 | **0.616** |

C: Concern; N, Necessity; SA: Statin adherence, eHL: eHealth literacy

For multicollinearity analysis (Table S5), all the indicators have a VIF value <5.0 except eHL3 with a VIF 6.620, which is still within the acceptable range of <10. Therefore, there was no significant multicollinearity among the indicators.

**Table S5. Multicollinearity analysis**

| **Construct domain** | **Indicator** | **VIF** |
| --- | --- | --- |
| Concern | C1 | 1.583 |
|  | C2 | 1.613 |
|  | C3 | 1.287 |
|  | C4 | 1.616 |
|  | C5 | 1.671 |
|  | C6 | 1.418 |
| Necessity | N1 | 1.684 |
|  | N2 | 2.119 |
|  | N3 | 2.094 |
|  | N4 | 1.959 |
|  | N5 | 1.118 |
| Statin adherence | SA1 | 3.103 |
|  | SA2 | 3.160 |
|  | SA3 | 2.886 |
|  | SA4 | 1.768 |
|  | SA5 | 1.440 |
|  | SA6 | 3.463 |
|  | SA7 | 2.433 |
|  | SA8 | 4.040 |
|  | SA9 | 2.446 |
|  | SA10 | 2.568 |
|  | SA11 | 2.845 |
|  | SA12 | 2.734 |
| eHealth Literacy | eHL1 | 3.083 |
|  | eHL2 | 4.888 |
|  | eHL3 | 6.620 |
|  | eHL4 | 3.864 |
|  | eHL5 | 3.512 |
|  | eHL6 | 2.383 |
|  | eHL7 | 2.541 |
|  | eHL8 | 1.868 |

**Formative model assessment**

Results of the formative model assessment were done using outer loading and outer weight (Table S6). According to these results, 3 indicators (Facebook, Google, WhatsApp) out of 4 indicators from the online source construct were removed because they had no significant contribution to the online source construct (outer weight was insignificant and outer loading <0.50). These indicators were removed from the subsequent final path analysis. For offline source, all 3 indicators were included in the offline resource construct as their outer loading was>0.50. Sources of information with a very small frequency were excluded in the formative model assessment, i.e., Instagram (n=4), TikTok (n=10) and Newspapers (n=8).

**Table S6. Formative model assessment for online and offline source constructs.**

| **Construct** | **Indicators** | **Outer loading** | **p-values** | **Outer weight** | **p-values** | **VIF** |
| --- | --- | --- | --- | --- | --- | --- |
| Online source | Google | -0.090 | 0.813 | -0.218 | 0.622 | 1.057 |
|  | **YouTube** | **0.963** | 0.085 | 0.979 | 0.101 | 1.024 |
|  | Facebook | 0.236 | 0.449 | 0.153 | 0.656 | 1.024 |
|  | WhatsApp | 0.018 | 0.959 | 0.056 | 0.883 | 1.058 |
| Offline source | **Friends** | **0.664** | 0.141 | 0.522 | 0.277 | 1.068 |
|  | **Family** | **0.540** | 0.095 | 0.341 | 0.336 | 1.073 |
|  | **HCP** | **0.730** | 0.033 | 0.643 | 0.090 | 1.017 |

HCP, healthcare professionals; VIF, variance inflation factor

**Structural model**

**Path analysis of confounding factors**

**Table S7. Path analysis between confounding factors and statin adherence**

| **Confounding factors** | **Statin adherence** | | | |
| --- | --- | --- | --- | --- |
|  | **β** | **SE** | **T statistics** | **P-values** |
| Age | -0.004 | 0.085 | 0.047 | 0.962 |
| Male | 0.150 | 0.142 | 1.058 | 0.290 |
| **Chinese** | 0.213 | 0.158 | 1.346 | **0.178** |
| **Indian** | 0.306 | 0.175 | 1.746 | **0.081** |
| Educational level | -0.025 | 0.077 | 0.323 | 0.747 |
| Income | -0.032 | 0.074 | 0.432 | 0.666 |
| Duration of statin use | 0.041 | 0.076 | 0.538 | 0.590 |
| Statin side effects | 0.064 | 0.179 | 0.357 | 0.721 |
| Presence of CVD | 0.037 | 0.154 | 0.242 | 0.809 |
| CAM use | -0.219 | 0.237 | 0.922 | 0.357 |

CVD: cardiovascular disease, CAM: complementary alternative medicine, SE: standardised error

**Table S8.** **Path coefficients and p-values of direct effects on necessity and concern of statins and statin adherence**

| **Path** | **β** | **SE** | **T** | **P-values** | **95% CI** | |
| --- | --- | --- | --- | --- | --- | --- |
|  |  |  |  |  | **UL** | **UL** |
| **Path a** |  |  |  |  |  |  |
| OHI submission → Concern | -0.040 | 0.062 | 0.643 | 0.520 | -0.161 | 0.082 |
| Active seeking → Concern | 0.323 | 0.142 | 2.268 | **0.023** | 0.042 | 0.605 |
| Passive encounter → Concern | -0.096 | 0.155 | 0.621 | 0.535 | -0.400 | 0.208 |
| Online source → Concern | 0.011 | 0.063 | 0.17 | 0.865 | -0.121 | 0.127 |
| Offline source → Concern | -0.148 | 0.080 | 1.843 | 0.065 | -0.270 | 0.053 |
| OHI submission → Necessity | 0.001 | 0.065 | 0.019 | 0.985 | -0.116 | 0.141 |
| Active seeking → Necessity | -0.016 | 0.169 | 0.096 | 0.923 | -0.341 | 0.308 |
| Passive encounter → Necessity | 0.256 | 0.200 | 1.282 | 0.200 | -0.183 | 0.612 |
| Online source → Necessity | 0.052 | 0.098 | 0.529 | 0.597 | -0.189 | 0.202 |
| Offline source → Necessity | -0.073 | 0.095 | 0.771 | 0.441 | -0.231 | 0.128 |
| **Path b** |  |  |  |  |  |  |
| Concern→ Statin adherence | -0.337 | 0.071 | 4.761 | **<0.001** | -0.485 | -0.209 |
| Necessity→ Statin adherence | 0.130 | 0.074 | 1.755 | 0.079 | -0.044 | 0.250 |
| **Path c** |  |  |  |  |  |  |
| OHI submission → Statin adherence | -0.042 | 0.130 | 0.324 | 0.746 | -0.332 | -0.203 |
| Active seeking → Statin adherence | -0.049 | 0.148 | 0.333 | 0.739 | -0.330 | 0.257 |
| Passive encounter → Statin adherence | -0.219 | 0.147 | 1.492 | 0.136 | -0.488 | 0.084 |
| Online source → Statin adherence | 0.151 | 0.124 | 1.210 | 0.226 | -0.081 | 0.420 |
| Offline source → Statin adherence | 0.057 | 0.104 | 0.550 | 0.582 | -0.189 | 0.240 |
| Chinese → Statin adherence | 0.244 | 0.144 | 1.700 | 0.089 | -0.029 | 0.526 |
| Indian **→** Statin adherence | 0.332 | 0.169 | 1.961 | **0.050** | -0.030 | 0.637 |

Path a: direct effect between independent variables and mediation variables, Path b: direct effect between mediating variables and dependent variables, Path c: direct effect between independent variables and dependent variables.

**Predictive power assessment**

**Table S9. R^2^ values for endogenous variables**

| **Construct** | **R^2^** |
| --- | --- |
| Concern | 0.042 |
| Necessity | 0.023 |
| Statin adherence | 0.251 |

# Table S10. Results of effect size f^2^ for three endogenous variables

| **Exogenous variables** | **Endogenous variables** | | |
| --- | --- | --- | --- |
|  | **Concern** | **Necessity** | **Statin adherence** |
| Active seeking OHI | 0.022 | 0.000 | 0.001 |
| Passive encounter OHI | 0.002 | 0.012 | 0.010 |
| OHI submission | 0.001 | 0.000 | 0.001 |
| Online source | 0.000 | 0.002 | 0.015 |
| Offline source | 0.022 | 0.005 | 0.004 |
| Chinese |  |  | 0.014 |
| Indian |  |  | 0.018 |
| Concern |  |  | 0.119 |
| Necessity |  |  | 0.021 |

**Mediation analysis**

**Table S11. Test of indirect effects of mediation**

| **Path** | **ab (indirect)** | **SE** | **T value** | **p-value** |
| --- | --- | --- | --- | --- |
| Active seeking →Concern → Statin adherence | -0.109 | 0.055 | 1.976 | **0.048** |
| Active seeking → Necessity → Statin adherence | -0.002 | 0.023 | 0.093 | 0.926 |
| Passive encounter → Concern → Statin adherence | 0.032 | 0.055 | 0.587 | 0.558 |
| Passive encounter → Necessity → Statin adherence | 0.033 | 0.032 | 1.029 | 0.303 |
| Online source → Concern → Statin adherence | -0.004 | 0.022 | 0.160 | 0.873 |
| Online source → Necessity → Statin adherence | 0.007 | 0.012 | 0.561 | 0.575 |
| Offline source → Concern → Statin adherence | 0.050 | 0.030 | 1.642 | 0.101 |
| Offline source → Necessity → Statin adherence | -0.010 | 0.014 | 0.705 | 0.481 |
| OHI submission → Concern → Statin adherence | 0.013 | 0.022 | 0.616 | 0.538 |
| OHI submission → Necessity → Statin adherence | 0.000 | 0.009 | 0.019 | 0.985 |

**Moderation analysis**

**Table S12. Test of moderating effects of trust in doctors and eHealth literacy on the relationship between OHI-seeking behaviour and statin adherence**

| **Paths** | **β** | **SE** | **T value** | **P Values** |
| --- | --- | --- | --- | --- |
| Trust in doctor x OHI submission → SA | -0.052 | 0.173 | 0.304 | 0.761 |
| Trust in doctor x Active seeking → SA | -0.092 | 0.151 | 0.607 | 0.544 |
| Trust in doctor x Passive encounter → SA | 0.017 | 0.187 | 0.090 | 0.928 |
| Trust in doctor x Online source → SA | -0.041 | 0.073 | 0.555 | 0.579 |
| eHealth literacy x OHI submission → SA | 0.106 | 0.259 | 0.409 | 0.683 |
| eHealth literacy x Active seeking → SA | -0.110 | 0.200 | 0.550 | 0.582 |
| eHealth literacy x Passive encounter → SA | -0.073 | 0.156 | 0.470 | 0.639 |
| eHealth literacy x Online source → SA | -0.047 | 0.158 | 0.294 | 0.769 |

SA, Statin adherence; OHI, online health information
